# Correlates of calf cramp in children with Charcot-Marie-Tooth disease

**DOI:** 10.1186/1757-1146-5-S1-O16

**Published:** 2012-04-10

**Authors:** Fiona E Blyton, Monique M Ryan, Robert A Ouvrier, Joshua Burns

**Affiliations:** 1 Discipline of Paediatrics and Child Health, The University of Sydney, Westmead, NSW, 2145, Australia; 2 Podiatry Program, The University of Newcastle, Ourimbah, 2258, Australia; 3 Neurosciences Department, Royal Children’s Hospital, Parkville, 3052, Victoria; 4 Institute for Neuroscience and Muscle Research, The Children’s Hospital at Westmead, Sydney, NSW, 2145, Australia

## Background

Leg muscle cramps have been identified as the strongest independent predictor of worse quality of life in Australian children with Charcot-Marie-Tooth disease Type 1A (CMT1A) [1]. There is no accepted treatment for cramp in children with CMT and the cause of cramp is not well understood. Potential therapeutic targets should be carefully identified to direct clinical trials of interventions.

## Materials and methods

81 children aged 2-16 years with CMT1A were recruited nationally through the Australasian Paediatric Charcot-Marie-Tooth Disease Registry [2]. Body size and measures of strength, ankle range, foot posture, balance, agility, endurance, gait and neurophysiology were assessed at The Children's Hospital at Westmead (Sydney) and Royal Children's Hospital (Melbourne). Data analysis included Pearson product moment and Spearman rank correlation coefficients for normally and non-normally distributed continuous data respectively, and Fischer's exact test for dichotomous data. Logistic regression analyses were performed to identify independent predictors of calf cramp.

## Results

Of the 81 children, 26 (32%) reported calf cramp and one child each reported toe, quadriceps or arm cramp. Calf cramp was associated (p < 0.05) with older age; presence of hand tremor; stronger foot inversion, eversion, dorsiflexion and plantarflexion; and better performance in long jump and 9-hole peg tests. Logistic regression analysis revealed only increasing age (OR 1.32; 95% CI: 1.11 to 1.58; p = 0.002) and presence of hand tremor (OR 3.81; 95% CI: 1.18 to 12.56; p = 0.028) as independent predictors of calf cramp.

## Conclusions

Calf cramps are common in children with CMT1A and worsen with age. This study revealed a previously unrecognised link between cramp and hand tremor in children with CMT1A. Further investigation of proposed mechanisms and risk factors common to both cramp and tremor will contribute to our understanding of these common complications of CMT1A.
